# Agent-based modeling of malaria control through mosquito aquatic habitats management in a traditional sub-Sahara grouping

**DOI:** 10.1186/s12889-020-10150-4

**Published:** 2021-03-11

**Authors:** Paul Layie, Vivient Corneille Kamla, Jean Claude Kamgang, Yves Emvudu Wono

**Affiliations:** 1 Department of Mathematics and Computer Science, Faculty of Science, University of Ngaoundere, Ngaoundere, Cameroon; 2 Departments of Mathematics and Computer Science, ENSAI, University of Ngaoundere, Ngaoundere, Cameroon; 3 Departments of Mathematics and Computer Science, University of Yaounde 1, Yaounde, Cameroon

**Keywords:** Malaria, Grouping, Aquatic habitat, Management, ABM

## Abstract

**Background:**

Africans pour dirty water around their houses which constitutes aquatic habitats (AH). These AH are sought by mosquitoes for larval development. Recent studies have shown the effectiveness of destroying AH around houses in reducing malaria incidence. An agent-based model is proposed for controlling malaria’s incidence through population sensitizing campaigns on the harmful effects of AH around houses.

**Methods:**

The environment is constituted of houses, AH, mosquitoes, humans, and hospital. Malaria’s spread dynamic is linked to the dynamics of humans and mosquitoes. The mosquito’s dynamic is represented by egg-laying and seeking blood. The human’s dynamic is animated by hitting mosquitoes. AH are destroyed each time by 10% of their starting number. The number of infected humans varied from 0-90 which led to a total of 1001 simulations.

**Results:**

When the number of houses and AH is equal, the results are approximate as the field data. At each reduction of AH, the incidence and prevalence tend more and more towards 0. When there is no AH and infected humans, the prevalence and incidence are at 0.

**Conclusions:**

When there is no AH site, the disease disappears completely. Global destruction of AH in an environment and using many parameters in the same model are recommended.

## Background

Malaria is one of the most dangerous infectious diseases in humans especially in Africa [1]. According to the World Health Organization (W.H.O.), malaria causes a lot of damage today with more than 219 million cases in 2017 worldwide, the majority of cases come from Sub-Saharan Africa [2]. For example, according to this report, we can note that 15 countries in sub-Saharan Africa and India have concentrated almost 80% of the total number of malaria cases in the world. The 10 countries where malaria is most prevalent in Africa are mostly sub-Saharan Africa. For mortality, Africa alone had obtained 93% of the malaria-related deaths with the majority of cases coming from sub-Saharan Africa. Economically, the governments of endemic countries have invested the US <DOLLAR/> 3.1 billion for the elimination and control of malaria in 2017. 3/4 of this amount has been invested in Africa, which is mostly sub-Saharan countries.

Beguin et al. [3] have defined the different types of sub-Saharan Africa grouping that can be encountered in Africa. According to them, a sub-Saharan Africa grouping is the fundamental cell of the family. So, when the family becomes too large or when the hope of an easier life pushes to divide, a new chief detaches from the family group and goes to establish his new home in other places, sometimes very distant (often hundreds of kilometers). The family can grow and become another grouping. These kinds of habitats are found mostly in Africa country and there are a small grouping and huge grouping [3, 4].

Among the most widely used malaria control measures in Africa, we find mainly: larval source management (LSM) and insecticide-treated nets (ITN) [4]. We note that despite all the efforts made to reduce or eradicate this disease, it continues to persist. Therefore it is important to bring new tools to fight against malaria. However, LSM notably played a very large role in reducing the incidence of malaria [5]. Among LSM methods, targeted source reduction (TSR) is the one that researchers precaunize much more than others [4, 5]. Indeed, according to several recent studies, TSR is more precaunized because it is more effective and less expensive [4]. However, TSR is contraried because they just treat a part of the AH while neglecting the other in a given environment. However, neglected other AH can make malaria reappear [5] and the disease will persist in the area. By knowing the number and speed of mosquito reproduction [4], even if there are few mosquitoes in an area, in a short time the number of mosquitoes will grow at a rapid rate. Do not also forget that mosquito can fly up to 10 - 12 km overflight during its lifetime with a speed of 1.6 - 2.4 km/h [6] and can live up to 40 days [7]. Depending on the houses of humans, larval development may be accentuated thereby increasing the number of mosquitoes. However, for the control of malaria, TSR are increasingly recommended. We can see that TSR is not a better method for Africans, since wastewater is a potential breeding ground for mosquitoes. This wastewater also comes out every day because it is part of actions linked to the habits of Africans.

Today, environmental management (EM) has taken Aquatic Habitat Management (AHM) of mosquitoes from new angles. First, new EM tools are being implemented and are costing more and more less [8]. We could for example mention aerial spraying through drones or planes [9, 10]. Thus, rather than going into the field to spray, we can use aerial rain by using products that are not harmful to humans such as new formulas of pesticides or biopesticides for example [10, 11]. However, the side effects on humans of spray products pose a major challenge. We realize that treating an entire area can become cheaper and take less time compared to going down on the ground to spray or destroy aquatic habitats of mosquitoes [12]. The overall treatment of an area requires a good understanding of the production and effectiveness of control measures for the entire area [13]. Second, communication tools can be used to sensitize the population and also promote environmental sanitation to avoid the production of AH as much as possible around houses [14–16]. Reduce the breeding grounds can prolong the development of the gonotrophic cycle and the spread of malaria [4]. The life cycle of the female anopheles consists of two phases: a laying phase (the mosquito searches for a lava site for the laying of eggs) and development and a phase of searching for blood in a host. Search oviposition sites and hosts can have important implications for the spread of malaria. The female mosquito cycle (egg-laying and blood-feeding) can help us to deny mosquito to have an opportunity to reduce the blood feeding’s frequency and egg-laying by using these programs. So, mortality over the cycle can be increase. Understanding the dynamic of mosquito help the programs of intervention’s development especially to know demarcate, and guide the perimeter of environmental sanitation areas. Nevertheless, taking parameters such as behaviors, movements, gonotrophic cycle, a cycle of malaria in each individual, and the status of individuals in the same model is often not possible. However, the consideration of these parameters in a model is important because it better reflects reality [7].

In the literature, some studies which have found good results using few parameters could have found other information if some other parameters had been considered. This is the case for example of Gu et al. [4] who have shown that Targeted Source Reduction is better than overall environmental management. According to Arifin et al. [5], they would have new information and more precise results if certain assumptions and/or parameters had been modified or taken into account. Among these parameters, there are for example the disposition of landscapes using absorbing and non-absorbing boundaries, the disposition of houses, and AH [5]. Also, Arifin et al. [5] reproached Gu et al. [4] for not running several simulations for the model. To resolve the problems of gu’s et al. study, Arifin et al. ([5]) replicated the results of [4], used a landscape generator tool, and replicated 1,800 simulations using non-absorbing and absorbing boundaries.

At the end of their work, Arifin et al. [5] mentioned that TSR would not help in malaria control like overall environmental management. Note that they just said it without providing any concrete proof. In this work, we will show that with real proof.

So far in the literature, no work simulates both: the activities and behavior, life cycle, status, development, and movement of each individual in the heterogeneous landscapes of sub-Saharan Africa grouping. In this work, we develop an ABM (Agent-Based Model) to simulate development and malaria evolution in each individual. Hence, our study’s objective was to assess and examine the impact of mosquito’s aquatic habitat destruction program and also bring out guidelines for LSM by aquatic habitat destruction. To achieve that, we take into account: the questing resting to properly define the activities of mosquitoes, the different stages of the disease to know the evolution of the disease in each individual, the different stages of mosquito development namely the immature (egg, larva, pupa) and adult phase to follow the individual development of individuals, the daily activities and behaviors of individuals (human and mosquitoes). We also take into account the efficiency of the distribution of mosquito AH and use the GIS (geographic information system) of sub-Saharan Africa grouping.

The mosquito development cycle is closely related to the evolution of plasmodium in each mosquito. A single bite is not always enough for a mosquito or human to become infectious. Yet most ABMs designed so far consider that a single mosquito bite can transmit plasmodium. Also, the majority of ABMs do not take into account the evolutionary cycle of malaria which would take us from susceptible mosquitoes to latent then infectious and from humans susceptible to recovery via latent and infectious. In this model, we use the questing resting as in Kamgang et al. [17] to represent the number of infectious bites. Also, we use the classes of individuals from the kamgang et al. and Chinit [18] mathematical models to represent the evolution of malaria in each individual.

Representing the number of infectious bites and the evolution of malaria in each individual is complicated because the movements of mosquito are dependent on each status. Although the agent-based models represent the behaviors and habits of each agent individually, it is nevertheless difficult to realize certain phenomena of epidemiology such as the number of infectious bites and the evolution of the disease in each individual. But today, with the development of computing, there are still ABM tools that we can use to represent real-world elements (which were difficult to achieve before) through complex behaviors.

## Methods

### Landscape

**Fig. 1:**
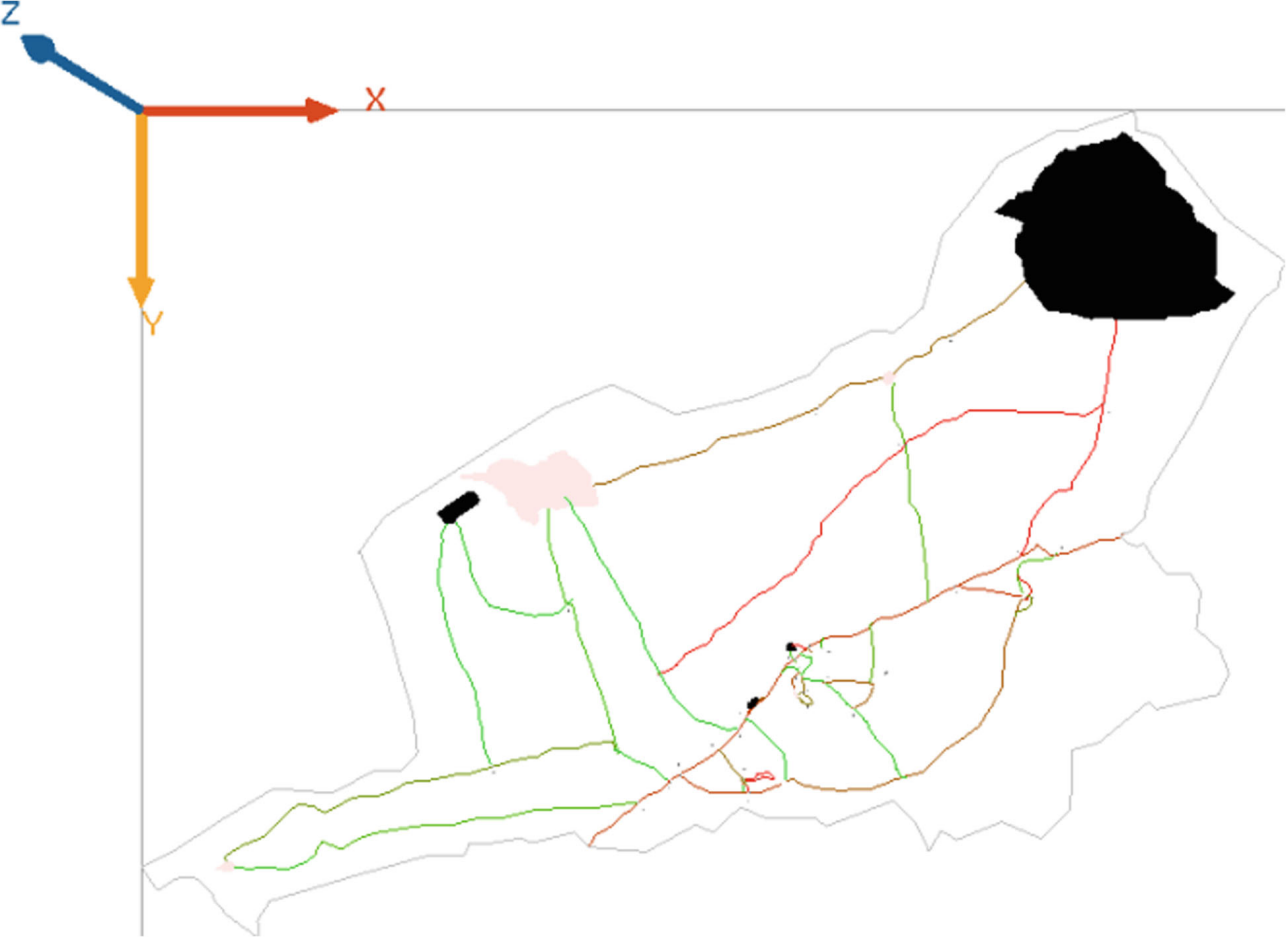
Demgoya grouping (comming from this study - made with QGIS open source software)

From the satellite image, we have circumscribed the Demgoya’s grouping (Fig. 1) in Cameroon, then target 30 houses. We transferred the data obtained into Qgis software before importing everything into the GAMA platform. We have also located 30 AH around each house, the distance of which varies between 1 m and 400 m from the associated house see Fig. 1. This reflects the environment and typical behavior of African villages [4]. Indeed, in African behavior, households generally pour dirty water not far from their homes which creates a small AH around the houses. Each house is inhabited by at least two humans and at most 5 humans as in [4]. As in [4], All aquatic habitats and houses were supposed to have the same mosquitoes’ attractiveness. The area of our grouping is estimated at 242.99 *km*^2^.

In this work, the research of the host and the egg-laying depend entirely on the position of mosquitoes, AH, humans, and houses. This is because the mosquito that is looking for the host (for the blood meal) searches the host or house closest. Also, after taking a blood meal, the mosquito searches for the nearest AH within its radius of flight for the laying.

### From eggs to immature adult mosquitoes

Since mosquitoes have four phases (one adult phase and three aquatic phases: egg, larva, lymph) of development [7], we model the transition from one phase to another according to a certain constant rate (Table 1). We set the maximum laying threshold at 5000 eggs/habitat as done by Gu et al. [4]. We set the eggs, larva, and lymph to 0 at each initialization. At each simulation step, when an adult mosquito has all its resources for egg-laying, it lays between 1-80 eggs per oviposition in a nest chosen from among those within its range. The migration of mosquitoes is not taken into account. This laying depends on the probability of laying which is also constant. We also fix the death probabilities of eggs, larvae, pupa, and adults (Table 1). After laying, the eggs have the possibility of becoming adult female mosquitoes after having gone through all the aquatic phases.

**Table 1:** The simulation parameters

Parameter	Value	Reference
Daily development rate of egg	0.3	[4]
Daily development rate of larva	0.2	[4]
Daily development rate of pupa	0.3	[4]
Daily mortality of immature stages	0.2	[4]
Fecundity	80 eggs/oviposition	[4]
Death rate of vectors due to questing activity	Compute	[17]
Incidence rate of infection for questing susceptible vectors	Compute	[17]
Incidence rate of successful blood meal for questing vectors	Compute	[17]
Transition rate from any resting state to a questing state	Compute	[17]
Rate at which resting vectors move to the questing state	Compute	[17]
Natural death rate of vectors	1/30	[17]
The per capita birth rate of humans	7,666∗10^−5^	[18]
The per capita birth rate of mosquitoes	0.4000	[18]
The per capita rate of progression of humans from the exposed state to the infectious state	8.333 * 10^−2^	[18]
The per capita recovery rate for humans from the infectious state to the recovered state	3.704 * 10^−3^	[18]
The per capita rate of loss of immunity for humans	1.460 * 10^−2^	[18]
The probability of transmission of infection from an infectious mosquito to a susceptible human given that a contact between the two occurs	0.8333	[18]
The probability of transmission of infection from a recovered (asymptomatic carrier) human to a susceptible mosquito given that a contact between the two occurs	8.333 * 10^−3^	[18]
The probability of transmission of infection from an infectious human to a susceptible mosquito given that a contact between the two occurs	2.000 * 10^−2^	[18]
Daily mortality of human	0.45* 10^−2^	This paper (data field)
The per capita disease-induced death rate for humans	0,62 * 10^−2^	This paper (data field)

### Adult mosquitoes

**Fig. 2:**
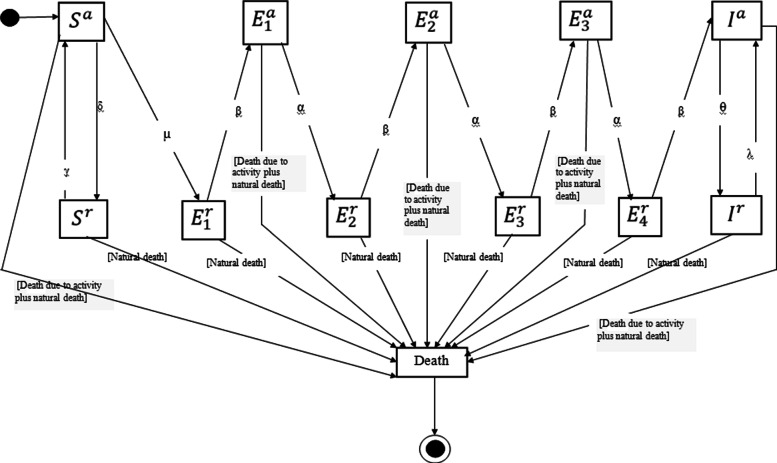
Mosquitoes infection statuses. Legends: *δ*: Become resting susceptible; *γ*: Become active susceptible; *μ*: Become resting latent 1; *β*: Become active latent and active infected; *α*: Become latent resting; *θ*: Become resting infected; *λ*: Become active infected. *S*_*a*_: active Susceptible, *S*_*r*_: resting susceptible, Active latent 1 (*Eva*_1_), Active latent 2 (*Eva*_2_), Active latent 3 (*Eva*_3_), Resting latent 1 (*Evr*_1_), Latent at rest 2 (*Evr*_2_), Latent at rest 3 (*Evr*_3_), Latent at rest 4 (*Evr*_4_), infectious at rest (*I*_*r*_) and active Infectious (*I*_*a*_)

For each mosquito, we followed the movement and the life cycle as did GU et al. [4]. We also add for each adult mosquito the cycle of malaria development as in the compartment or mathematical models. In these models, we move from one class to another by rates and times known by epidemiologists. We use the same principle to model the cycle of malaria development in each mosquito. The transition from one class to another is function of a constant rate dependent on the dates of emergence and last blood feeding at each transition (Table 1). Note that all mosquitoes newly emerged as adults are considered susceptible. Mosquitoes leave the population through natural mortality or through additive mortality due to activity. We assume that any individual who crosses the boundary of the area is considered dead. To represent the number of infectious bites, we use the questing resting model of Kamgang et al. (Fig. 2). The mosquito performs a random flight within its radius of flight which is 250 m/day as in [4]. The mosquito’s hours of activity are between 6 p.m. and 6 a.m. [7]. Adult mosquitoes also have a probability of death depending on their class (see Table 1). The normal lifespan of a mosquito is estimated at 40 days [7].

### The human population

In African groups, there are generally not many inhabitants [7], that is why we have considered 30 houses. In these 30 houses, we find the human who spends the whole night (6 p.m. - 6 a.m.) to sleep. We also represent the development of malaria in each human using the compartments or classes of mathematical models in the literature. Deaths are represented by the probability depending on each class (see Table 1). We distinguish here men and women to be able to represent births, migration is not taken into account here. Indeed, when a woman is between 15 and 50 years old, she can give birth to one or two children. The maximum lifespan of a human being fixed to 100 years, at the start of each simulation we allocate an age of between 1 to 100 years for each human.

### Transmission and evolution’s cycle of malaria

We used susceptible-latent-infected-recovered (SEIR) compartment models to represent the dynamics of malaria transmission in the simulation. For this, we used the campartment models of Chinit [18] and Kamgang et al. [17] to represent the classes of human and mosquito individuals respectively. We have chosen the Chinit model for humans because it takes into account the majority of the individual’s classes including the return of the resettles to the susceptible class. The Kamgang et al. model was chosen for mosquitoes because it represents the majority of mosquito classes and takes into account questing-resting. Indeed, the questing resting makes it possible to represent the fact that the mosquito bites a human several times before becoming infected or infecting humans.

**Fig. 3:**
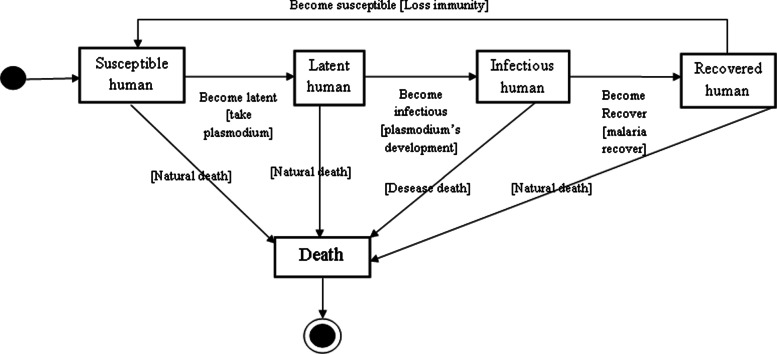
Human infection statuses

The classes of individuals of humans are: Susceptible (*s*_*h*_), Latent (*E*_*h*_), Infected (*I*_*h*_), Recovered (*R*_*h*_) (see Fig. 3). When a human is healthy, it is considered that he is susceptible and he becomes latent (or enters the incubation phase) when he receives an acceptable level of plasmodium represented here by a probability also called contact rate as in Chinits. Note that this is only valid when an infected and active mosquito comes into contact with a healthy human. After 15 days in this phase, humans become infectious and can transmit malaria to a healthy mosquito. It is at this level that he seeks the health center to treat himself. If after 100 days [4], he cannot find the health center for treatment (represented here by the cure rate), he dies of malaria, otherwise, he becomes recovered. Between one to three months [19], the recovered becomes susceptible again at a certain rate. The cycle can then start again and this happens on every human. At each initialization, each individual has a randomly assigned class date, that is, 1-100 years for humans and 1-40 days for mosquitoes.

The mosquito classes of individuals are made up of: Susceptible active Sa, susceptible resting (Mosquitoes rests either in a house or in an aquatic habitat) Sr, Active latent 1 (*Eva*_1_), Active latent 2 (*Eva*_2_), Active latent 3 (*Eva*_3_), Resting latent 1 (*Evr*_1_), Latent at rest 2 (*Evr*_2_), Latent at rest 3 (*Evr*_3_), Latent at rest 4 (*Evr*_4_), infectious at rest (which is not in motion) Ir and Infectious active Ia (see Fig. 2). Likewise, a healthy mosquito is considered as active susceptible if it searches of a resource (aquatic habitat or house) and susceptible at rest when not in motion. When an active susceptible comes into contact with an infected human or recovered, it becomes latent with a certain rate. If the blood dose is necessary for the development of these eggs, he can return to the AH to rest. This is the reason why the mosquito in the model starts from susceptible active to latent at rest. After a certain time (rest period represented here by a rate), the mosquito if it is still alive, switches from the *Er*_*i*_ class to the *Ea*_*i*_ class (i = 1,2,3). When a mosquito in the *Ea*_*i*_ class manages to take its blood meal (with speed), it migrates to the *Er*_*i*+1_ class. After a certain time spent in the Er4 class, it migrates to the Ia class and when it succeeds in having its blood meal, it switches from the Ia class to the Ir class; after he returns to class Ia and this repeats for life. For infection rates (mosquito to human infection and vice versa), we used Chinit rates. We take the infection rates of Chinit rather than that for Kamgng et al. because it represents the different links of infections (For example, in Kamgang et al. model there are no recovered but Chinit takes into account the recovered which can also transmit the disease.

### Managing the environment by destroying aquatic habitat

In our environment, we have identified 30 houses, 30 AH, and a health center. Throughout our study, the houses remained fixed apart from the AH which were varied each time. Indeed, our main objective was to destroy the AH of 10% of the number of starting AH to see the evolution of malaria and mosquitoes. The control of our environment has been covered on 30 AH first, then 27 AH, 24, 21, 18, 15, 12, 9, 6, 3, and finally 0 AH. In every initialization, we consider that each AH has on average 5 mosquitoes and these mosquitoes can increase (up to a maximum of 5000 per AH) and decrease (to a minimum of 0) depending on the cycle of life, behavior, activities, or movement and interactions between individuals. This is how at each initialization, when the environment has 30, 27, 24, 21, 18, 15, 12, 9, 6, 3 and 0 AH, we have 150, 135, 120, 105, 90, 75, 60, 45, 30, 15 and 0 mosquitoes respectively. we also vary the number of infected humans from 0 to 90. To be consistent with reality, we compared our results when there is no heel destruction with the results of the field data. Indeed, it is this scenario that reflects the reality on the ground. So by comparing these results, we don’t get a big difference.

### Simulation

For the simulation, we took 6 hours as a simulation step to be able to highlight the working hours of our individuals. Also, each simulation evolves over one year, which makes 1440 cycles per simulation. At each initialization of the simulations, we considered 90 humans (with an average of 3 inhabitants per house). But over time each house can have between 2 and 5 inhabitants. For mosquitoes, we considered an average of 5 mosquitoes per AH at initialization. This number varies over time due in particular to births and deaths.

In our simulation, we first take the same number of houses and AH and vary the number of infected humans to 0-90 and report the result. After, we destroy the AH with a rate of 10% of the number of starting AH until rich to 0 AH and vary every time infected human to 0-90. In the end, we had a total of 1001 simulations. When we vary the number of infected humans from 0 to 90, the distribution for other classes of humans does not respect any distribution rate because the interests class is the infected class. For mosquitoes, we distribute the Sa, Sr, Ia, and Ir equitably and each class is equal to 13.33% of the initial number of mosquitoes in each simulation. Likewise, the latent classes (*Eva*_1_,*Eva*_2_,*Eva*_3_,*Evr*_1_,*Evr*_2_,*Evr*_3_,*Evr*_4_) are also distributed equitably each time. Thus, each latent class represents 6.67% of the initial number of mosquitoes.

The simulation was performed in a machine with capacity: 500GB of DD, 4GB of RAM, and an Intel (R) Core (TM) i3-2310M, 2.1GHZ.

## Results

### Presentation of some curves

The following figures show the shape of some curves of the simulation. We just represent the curves of the simulation in the cases where we have: 0 infected human (and 0%, 10%, 50%, 100% destruction of AH), 1 infected human (and 0%, 10%, 50%, 100% destruction of AH), 9 infected humans corresponding to 10% of the initial number of humans (and 0%, 10%, 50%, 100% destruction of AH), 45 infected humans corresponding to 50% of the initial number of humans (and 0%, 10%, 50%, 100% destruction of AH), 90 infected humans corresponding to 100% of the initial number of humans (and 0%, 10%, 50%, 100% destruction of AH). We also present some curves on the mosquito population, in particular in the following cases: 0 infected human and 10% AH destruction; 1 infected human, 10% and 100% AH destruction; 9 humans infected and 50% destruction of AH; 45 infected humans, 50% and 100% AH destruction.

### Interpretation of curves

**Fig. 4:**
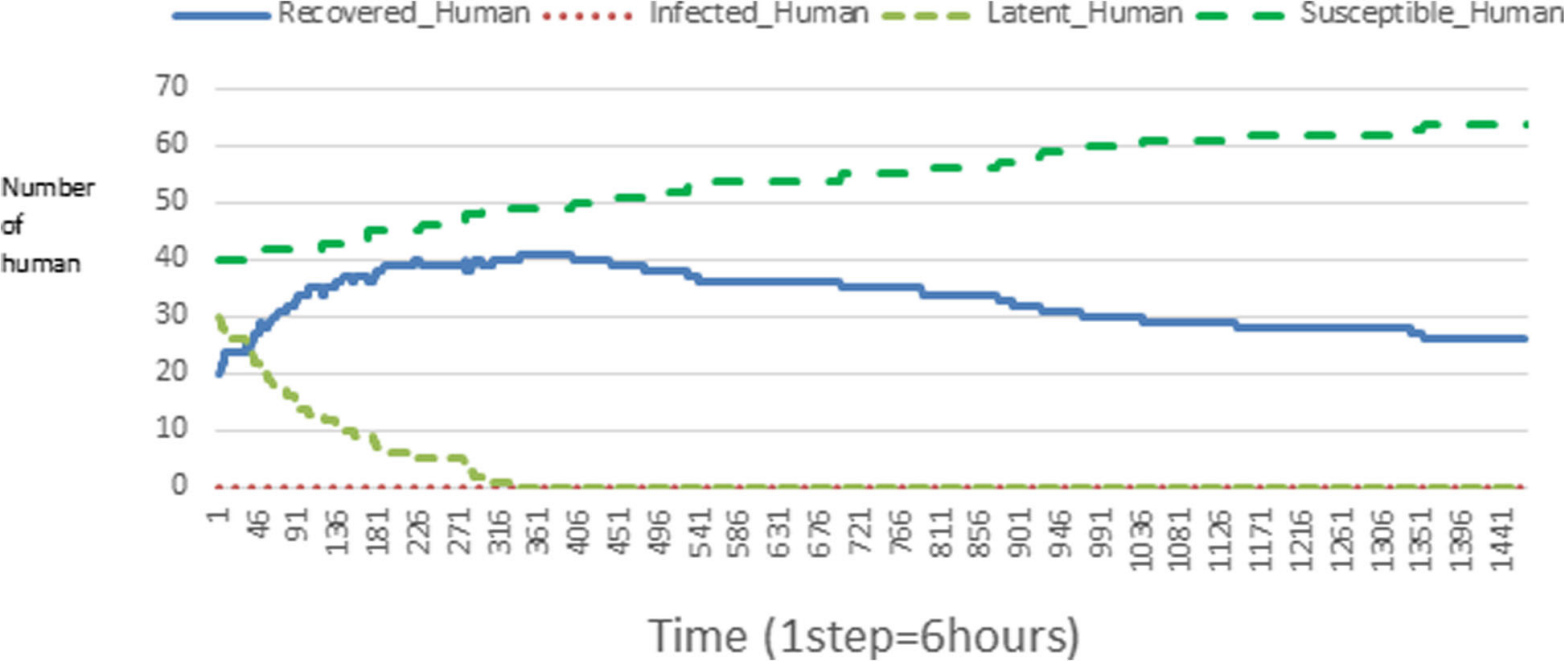
Human population: Evolution of susceptible, latent, infected, and recovery. The initial conditions are: number of infected human = 0, Number of AH = 0 *S*_*h*_ = 40, *E*_*h*_ = 30, *I*_*h*_ = 0, *R*_*h*_ = 20, *S*_*va*_ = 0, *S*_*vr*_ = 0, *E*_*va*1_ = 0, *E*_*va*2_ = 0, *E*_*va*3_ = 0, *E*_*vr*1_ = 0, *E*_*vr*2_ = 0, *E*_*vr*3_ = 0, *E*_*vr*4_ = 0, *I*_*va*_ = 0 and *I*_*vr*_ = 0

**Fig. 5:**
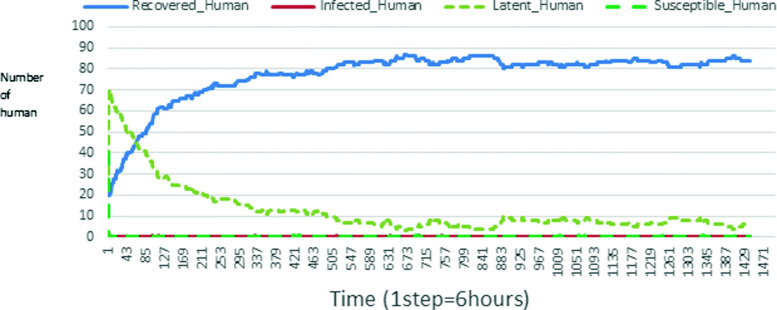
Human population: Evolution of susceptible, latent, infected, and recovery. The initial conditions are: number of infected human = 0, Number of AH = 3 *S*_*h*_ = 40, *E*_*h*_ = 30, *I*_*h*_ = 0, *R*_*h*_ = 20, *S*_*va*_ = 2, *S*_*vr*_ = 2, *E*_*va*1_ = 1, *E*_*va*2_ = 1, *E*_*va*3_ = 1, *E*_*vr*1_ = 1, *E*_*vr*2_ = 1, *E*_*vr*3_ = 1, *E*_*vr*4_ = 1, *I*_*va*_ = 2 and *I*_*vr*_ = 2

**Fig. 6:**
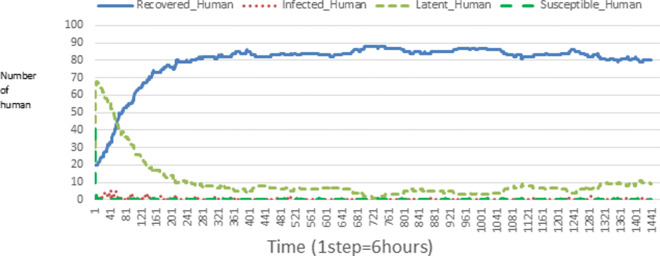
Human population: Evolution of susceptible, latent, infected, and recovery. The initial conditions are: number of infected human = 0, Number of AH = 15 *S*_*h*_ = 40, *E*_*h*_ = 30, *I*_*h*_ = 0, *R*_*h*_ = 20, *S*_*va*_ = 10, *S*_*vr*_ = 10, *E*_*va*1_ = 5, *E*_*va*2_ = 5, *E*_*va*3_ = 5, *E*_*vr*1_ = 5, *E*_*vr*2_ = 5, *E*_*vr*3_ = 5, *E*_*vr*4_ = 5, *I*_*va*_ = 10 and *I*_*vr*_ = 10

**Fig. 7:**
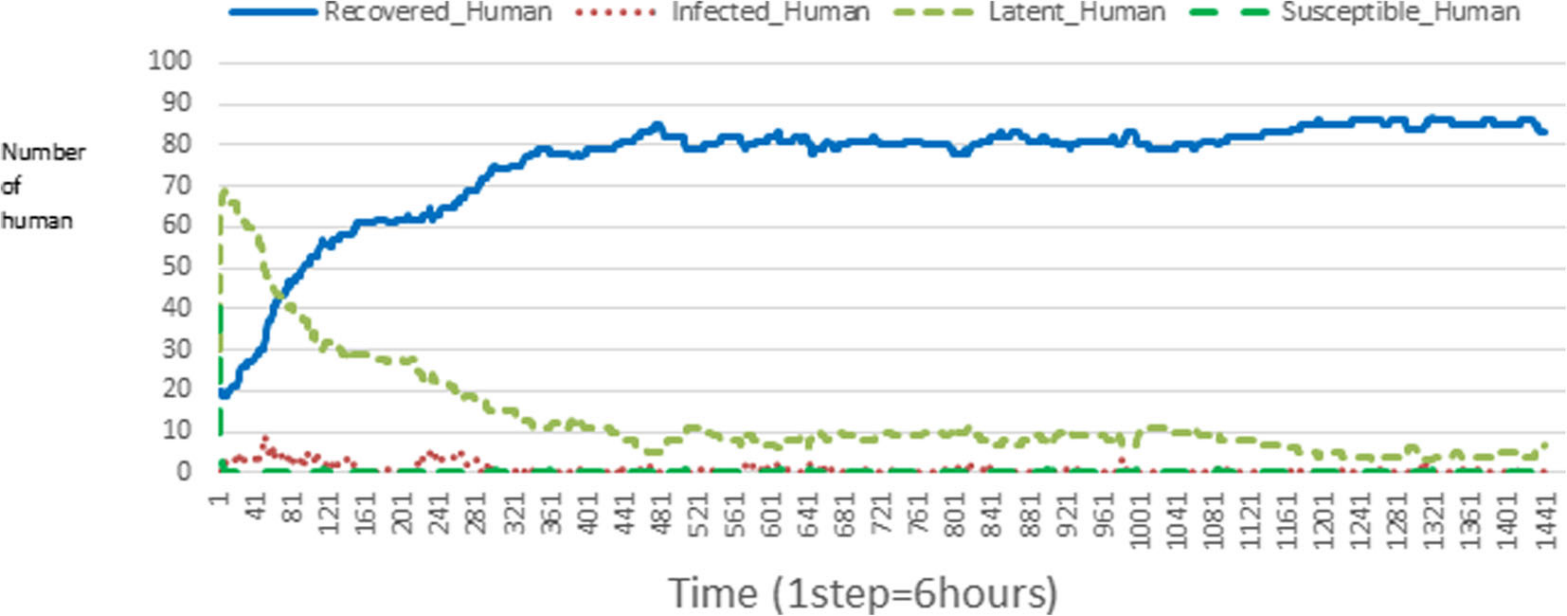
Human population: Evolution of susceptible, latent, infected, and recovery. The initial conditions are: number of infected human = 0, Number of AH = 30 *S*_*h*_ = 40, *E*_*h*_ = 30, *I*_*h*_ = 0, *R*_*h*_ = 20, *S*_*va*_ = 20, *S*_*vr*_ = 20, *E*_*va*1_ = 10, *E*_*va*2_ = 10, *E*_*va*3_ = 10, *E*_*vr*1_ = 10, *E*_*vr*2_ = 10, *E*_*vr*3_ = 10, *E*_*vr*4_ = 10, *I*_*va*_ = 20 and *I*_*vr*_ = 20

**Fig. 8:**
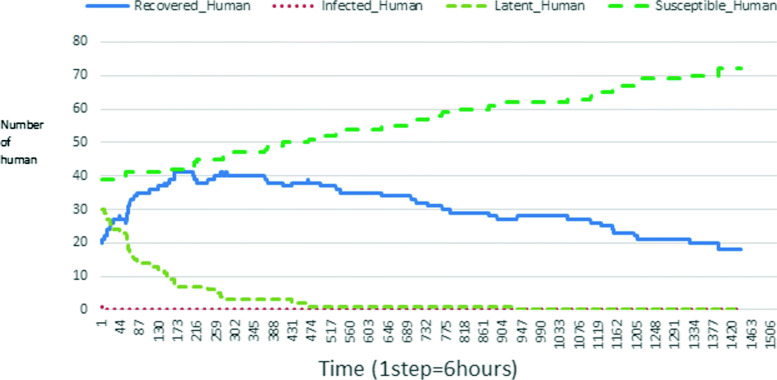
Human population: Evolution of susceptible, latent, infected, and recovery. The initial conditions are: number of infected human = 1, Number of AH = 0 *S*_*h*_ = 39, *E*_*h*_ = 30, *I*_*h*_ = 1, *R*_*h*_ = 20, *S*_*va*_ = 0, *S*_*vr*_ = 0, *E*_*va*1_ = 0, *E*_*va*2_ = 0, *E*_*va*3_ = 0, *E*_*vr*1_ = 0, *E*_*vr*2_ = 0, *E*_*vr*3_ = 0, *E*_*vr*4_ = 0, *I*_*va*_ = 0 and *I*_*vr*_ = 0

In Figs. 4, 5, 6, 7, and 8 there is no HA (i.e. the number of HA is equal to 0) which means that there are no mosquitoes in the environment. We observe in these figures that the state of health of the population tends more and more to be normal in the time. This means that all humans who were not susceptible change their status until to become susceptible. This is the reason why the likelihood curve grows over time. On the other hand, the curve of the other statuses (latent, infected, restored) decreases over time. Other things, we realize that when there are infected humans in the environment, they quickly change their status in other to be restored and then susceptible. This is what the humans’ infected curves decrease until to be at zero at a certain time. Once humans become susceptible, they remain there for the rest of their lives because there are no mosquitoes in the environment.

**Fig. 9:**
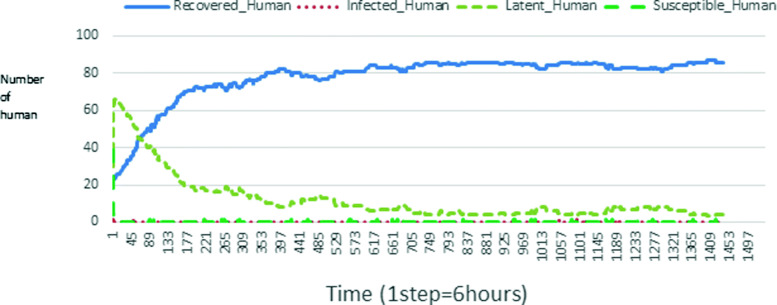
Human population: Evolution of susceptible, latent, infected, and recovery. The initial conditions are: number of infected human = 1, Number of AH = 3 *S*_*h*_ = 39, *E*_*h*_ = 30, *I*_*h*_ = 1, *R*_*h*_ = 20, *S*_*va*_ = 2, *S*_*vr*_ = 2, *E*_*va*1_ = 1, *E*_*va*2_ = 1, *E*_*va*3_ = 1, *E*_*vr*1_ = 1, *E*_*vr*2_ = 1, *E*_*vr*3_ = 1, *E*_*vr*4_ = 1, *I*_*va*_ = 2 and *I*_*vr*_ = 2

**Fig. 10:**
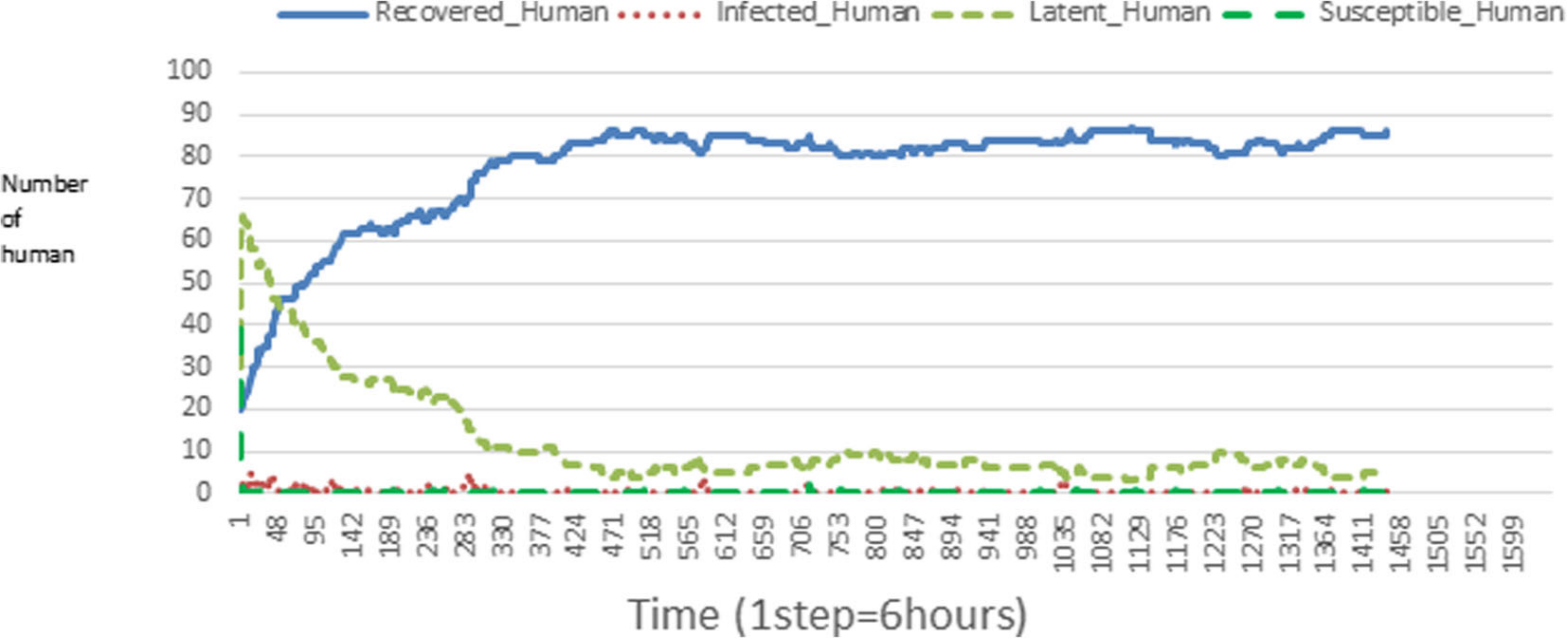
Human population: Evolution of susceptible, latent, infected, and recovery. The initial conditions are: number of infected human = 1, Number of AH = 15 *S*_*h*_ = 39, *E*_*h*_ = 30, *I*_*h*_ = 1, *R*_*h*_ = 20, *S*_*va*_ = 10, *S*_*vr*_ = 10, *E*_*va*1_ = 5, *E*_*va*2_ = 5, *E*_*va*3_ = 5, *E*_*vr*1_ = 5, *E*_*vr*2_ = 5, *E*_*vr*3_ = 5, *E*_*vr*4_ = 5, *I*_*va*_ = 10 and *I*_*vr*_ = 10

**Fig. 11:**
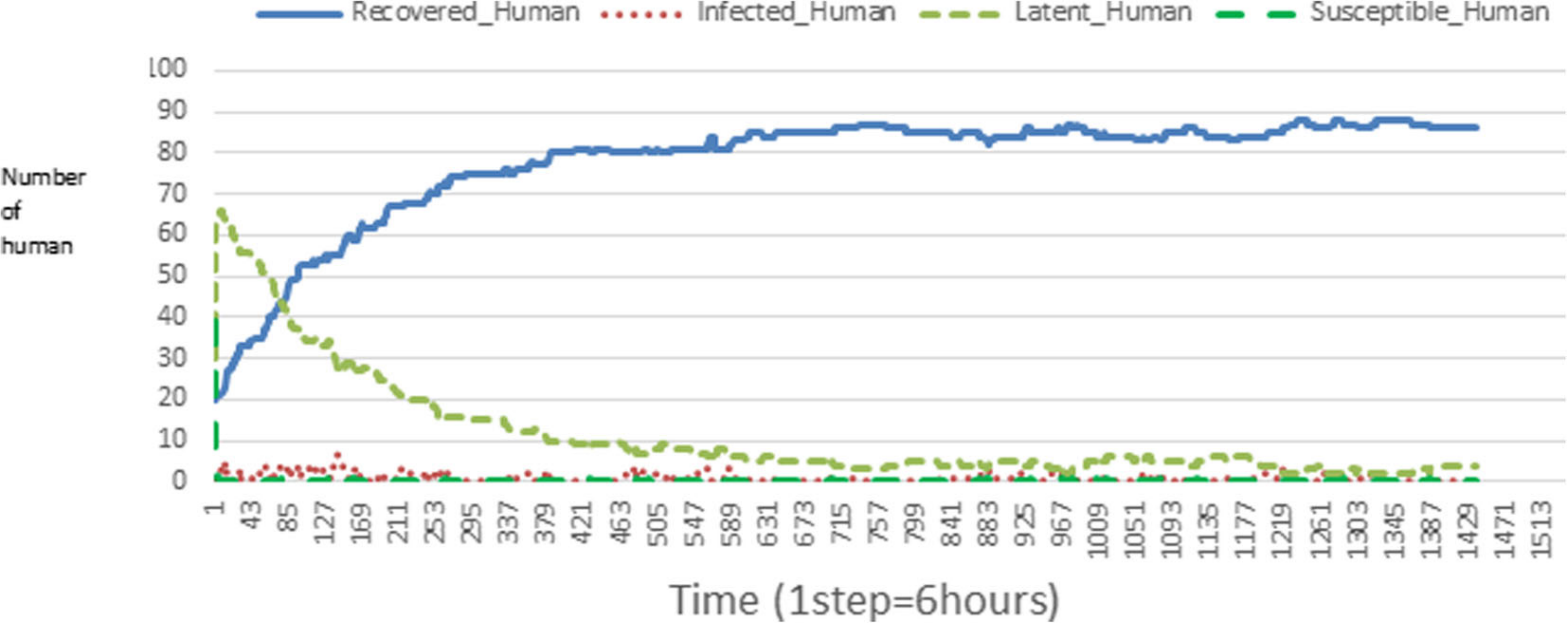
Human population: Evolution of susceptible, latent, infected, and recovery. The initial conditions are: number of infected human = 1, Number of AH = 30 *S*_*h*_ = 39, *E*_*h*_ = 30, *I*_*h*_ = 1, *R*_*h*_ = 20, *S*_*va*_ = 20, *S*_*vr*_ = 20, *E*_*va*1_ = 10, *E*_*va*2_ = 10, *E*_*va*3_ = 10, *E*_*vr*1_ = 10, *E*_*vr*2_ = 10, *E*_*vr*3_ = 10, *E*_*vr*4_ = 10, *I*_*va*_ = 20 and *I*_*vr*_ = 20

**Fig. 12:**
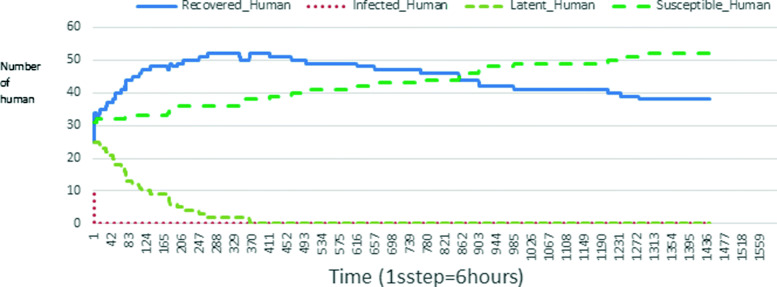
Human population: Evolution of susceptible, latent, infected, and recovery. The initial conditions are: number of infected human = 9, Number of AH = 0 *S*_*h*_ = 31, *E*_*h*_ = 25, *I*_*h*_ = 9, *R*_*h*_ = 25, *S*_*va*_ = 0, *S*_*vr*_ = 0, *E*_*va*1_ = 0, *E*_*va*2_ = 0, *E*_*va*3_ = 0, *E*_*vr*1_ = 0, *E*_*vr*2_ = 0, *E*_*vr*3_ = 0, *E*_*vr*4_ = 0, *I*_*va*_ = 0 and *I*_*vr*_ = 0

**Fig. 13:**
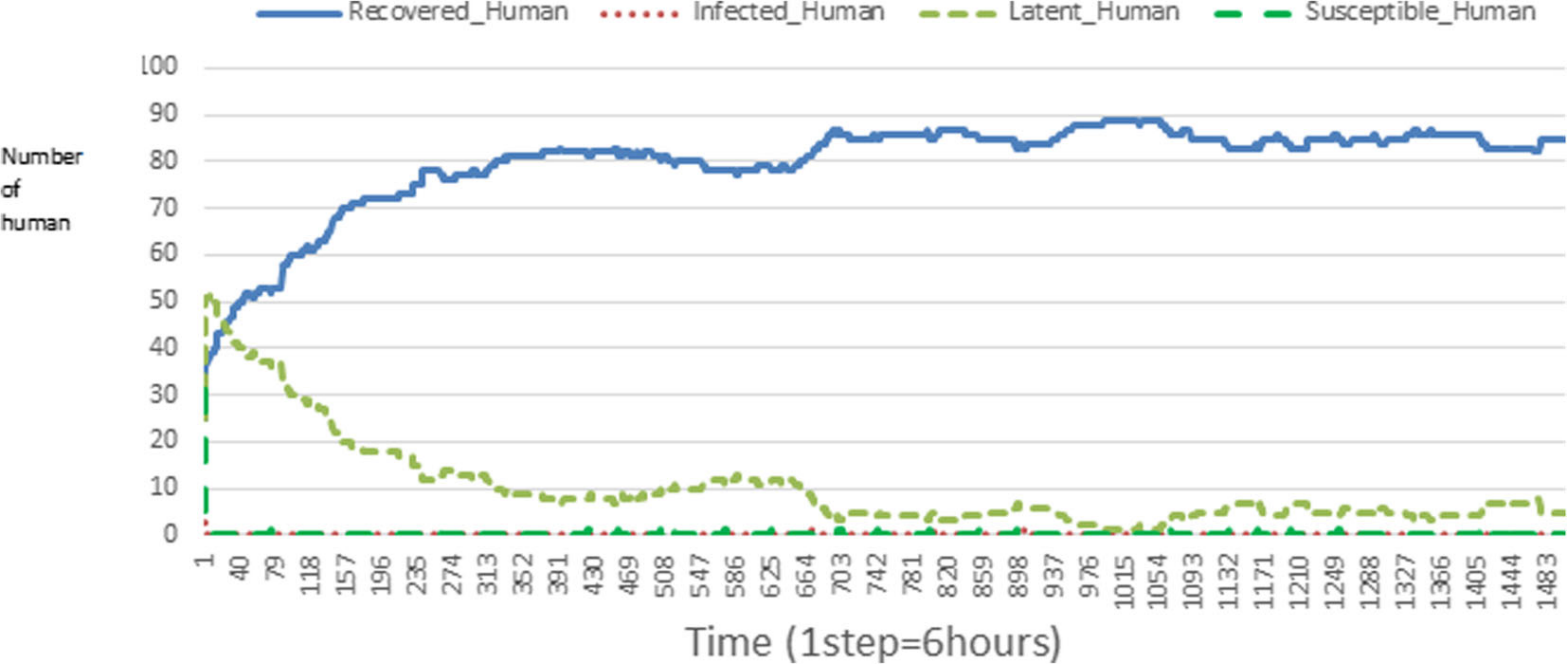
Human population: Evolution of susceptible, latent, infected, and recovery. The initial conditions are: number of infected human = 9, Number of AH = 3 *S*_*h*_ = 31, *E*_*h*_ = 25, *I*_*h*_ = 0, *R*_*h*_ = 25, *S*_*va*_ = 2, *S*_*vr*_ = 2, *E*_*va*1_ = 1, *E*_*va*2_ = 1, *E*_*va*3_ = 1, *E*_*vr*1_ = 1, *E*_*vr*2_ = 1, *E*_*vr*3_ = 1, *E*_*vr*4_ = 1, *I*_*va*_ = 2 and *I*_*vr*_ = 2

**Fig. 14:**
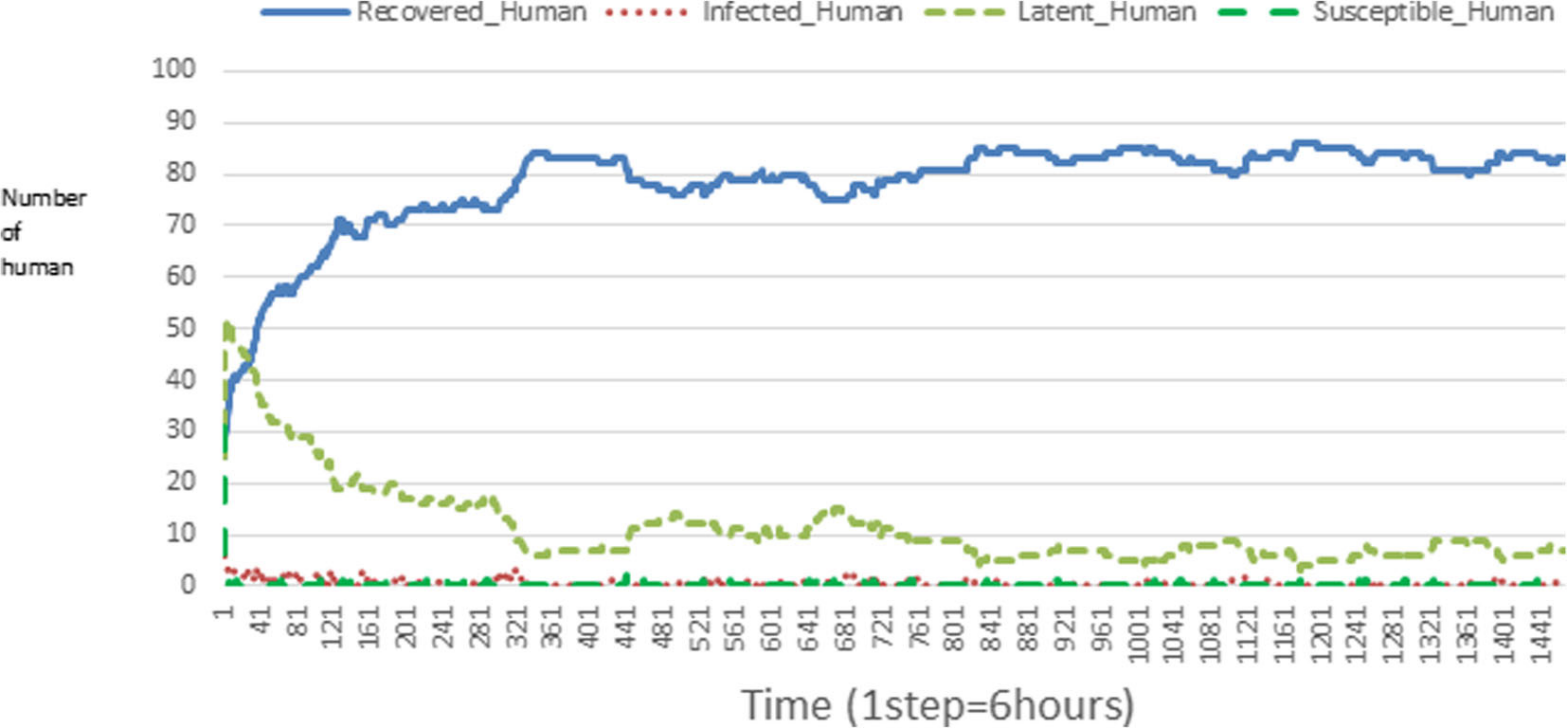
Human population: Evolution of susceptible, latent, infected, and recovery. The initial conditions are: number of infected human = 9, Number of AH = 15 *S*_*h*_ = 31, *E*_*h*_ = 25, *I*_*h*_ = 0, *R*_*h*_ = 25, *S*_*va*_ = 10, *S*_*vr*_ = 10, *E*_*va*1_ = 5, *E*_*va*2_ = 5, *E*_*va*3_ = 5, *E*_*vr*1_ = 5, *E*_*vr*2_ = 5, *E*_*vr*3_ = 5, *E*_*vr*4_ = 5, *I*_*va*_ = 10 and *I*_*vr*_ = 10

**Fig. 15:**
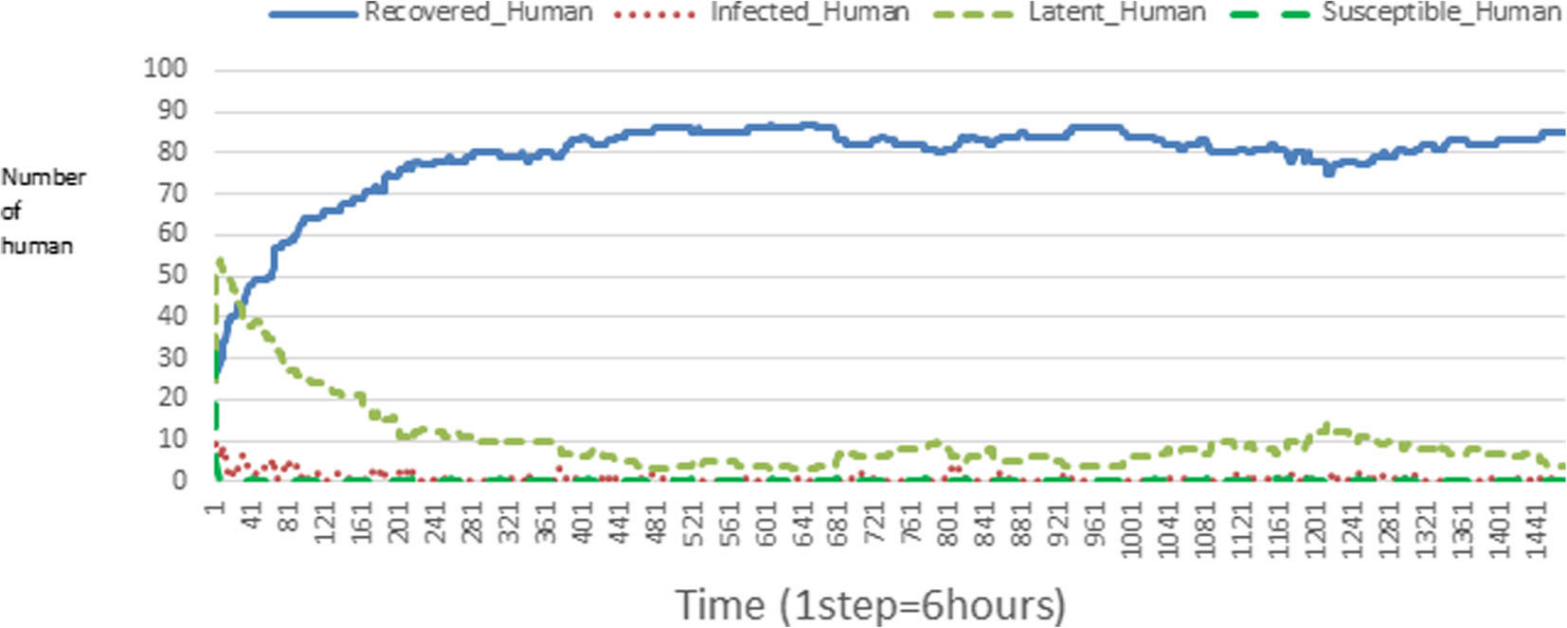
Human population: Evolution of susceptible, latent, infected, and recovery. The initial conditions are: number of infected human = 9, Number of AH = 30 *S*_*h*_ = 31, *E*_*h*_ = 25, *I*_*h*_ = 9, *R*_*h*_ = 25, *S*_*va*_ = 20, *S*_*vr*_ = 20, *E*_*va*1_ = 10, *E*_*va*2_ = 10, *E*_*va*3_ = 10, *E*_*vr*1_ = 10, *E*_*vr*2_ = 10, *E*_*vr*3_ = 10, *E*_*vr*4_ = 10, *I*_*va*_ = 20 and *I*_*vr*_ = 20

**Fig. 16:**
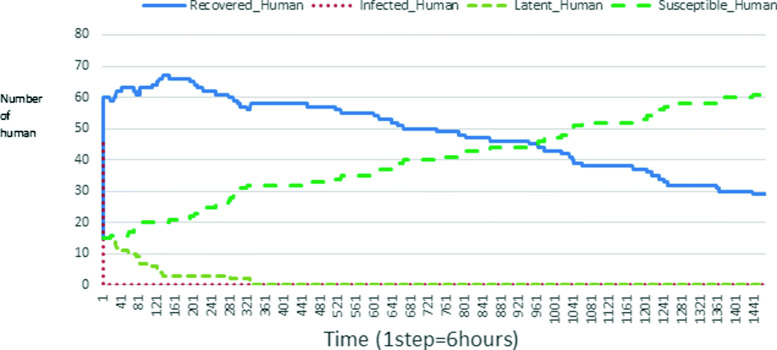
Human population: Evolution of susceptible, latent, infected, and recovery. The initial conditions are: number of infected human = 45, Number of AH = 0 *S*_*h*_ = 15, *E*_*h*_ = 15, *I*_*h*_ = 45, *R*_*h*_ = 15, *S*_*va*_ = 0, *S*_*vr*_ = 0, *E*_*va*1_ = 0, *E*_*va*2_ = 0, *E*_*va*3_ = 0, *E*_*vr*1_ = 0, *E*_*vr*2_ = 0, *E*_*vr*3_ = 0, *E*_*vr*4_ = 0, *I*_*va*_ = 0 and *I*_*vr*_ = 0

**Fig. 17:**
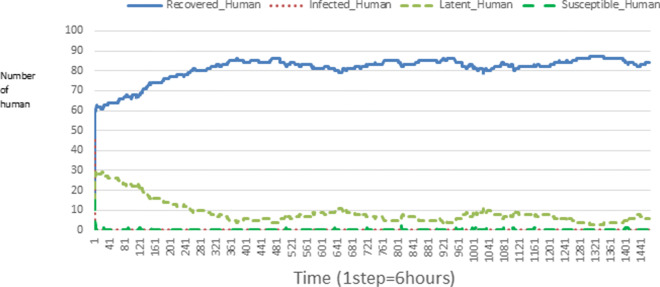
Human population: Evolution of susceptible, latent, infected, and recovery. The initial conditions are: number of infected human = 45, Number of AH = 3 *S*_*h*_ = 15, *E*_*h*_ = 15, *I*_*h*_ = 45, *R*_*h*_ = 15, *S*_*va*_ = 2, *S*_*vr*_ = 2, *E*_*va*1_ = 1, *E*_*va*2_ = 1, *E*_*va*3_ = 1, *E*_*vr*1_ = 1, *E*_*vr*2_ = 1, *E*_*vr*3_ = 1, *E*_*vr*4_ = 1, *I*_*va*_ = 2 and *I*_*vr*_ = 2

**Fig. 18:**
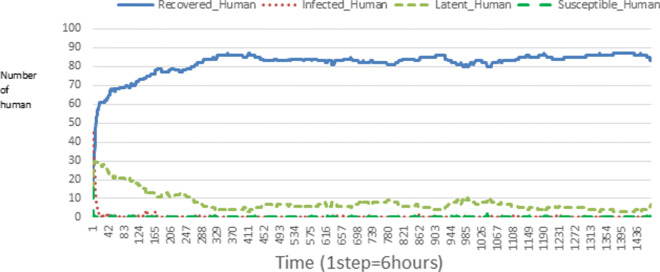
Human population: Evolution of susceptible, latent, infected, and recovery. The initial conditions are: number of infected human = 45, Number of AH = 15 *S*_*h*_ = 15, *E*_*h*_ = 15, *I*_*h*_ = 45, *R*_*h*_ = 15, *S*_*va*_ = 10, *S*_*vr*_ = 10, *E*_*va*1_ = 5, *E*_*va*2_ = 5, *E*_*va*3_ = 5, *E*_*vr*1_ = 5, *E*_*vr*2_ = 5, *E*_*vr*3_ = 5, *E*_*vr*4_ = 5, *I*_*va*_ = 10 and *I*_*vr*_ = 10

**Fig. 19:**
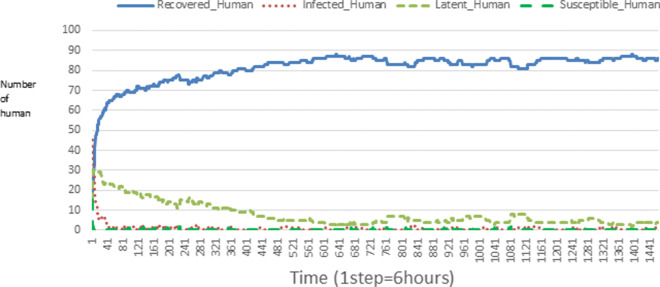
Human population: Evolution of susceptible, latent, infected, and recovery. The initial conditions are: number of infected human = 45, Number of AH = 30 *S*_*h*_ = 15, *E*_*h*_ = 15, *I*_*h*_ = 45, *R*_*h*_ = 15, *S*_*va*_ = 20, *S*_*vr*_ = 20, *E*_*va*1_ = 10, *E*_*va*2_ = 10, *E*_*va*3_ = 10, *E*_*vr*1_ = 10, *E*_*vr*2_ = 10, *E*_*vr*3_ = 10, *E*_*vr*4_ = 10, *I*_*va*_ = 20 and *I*_*vr*_ = 20

**Fig. 20:**
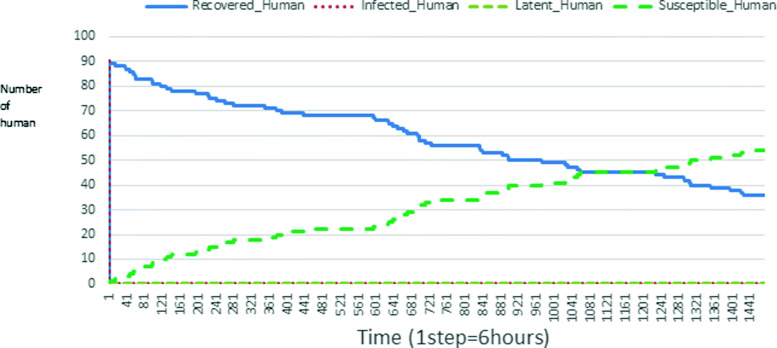
Human population: Evolution of susceptible, latent, infected, and recovery. The initial conditions are: number of infected human = 90, Number of AH = 0 *S*_*h*_ = 0, *E*_*h*_ = 0, *I*_*h*_ = 90, *R*_*h*_ = 0, *S*_*va*_ = 0, *S*_*vr*_ = 0, *E*_*va*1_ = 0, *E*_*va*2_ = 0, *E*_*va*3_ = 0, *E*_*vr*1_ = 0, *E*_*vr*2_ = 0, *E*_*vr*3_ = 0, *E*_*vr*4_ = 0, *I*_*va*_ = 0 and *I*_*vr*_ = 0

**Fig. 21:**
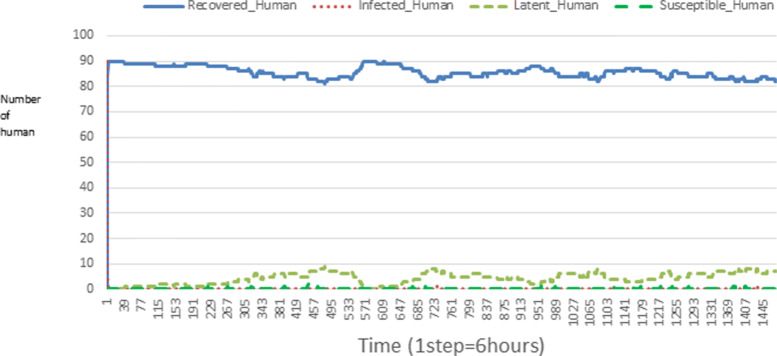
Human population: Evolution of susceptible, latent, infected, and recovery. The initial conditions are: number of infected human = 90, Number of AH = 3 *S*_*h*_ = 0, *E*_*h*_ = 0, *I*_*h*_ = 90, *R*_*h*_ = 0, *S*_*va*_ = 2, *S*_*vr*_ = 2, *E*_*va*1_ = 1, *E*_*va*2_ = 1, *E*_*va*3_ = 1, *E*_*vr*1_ = 1, *E*_*vr*2_ = 1, *E*_*vr*3_ = 1, *E*_*vr*4_ = 1, *I*_*va*_ = 2 and *I*_*vr*_ = 2

**Fig. 22:**
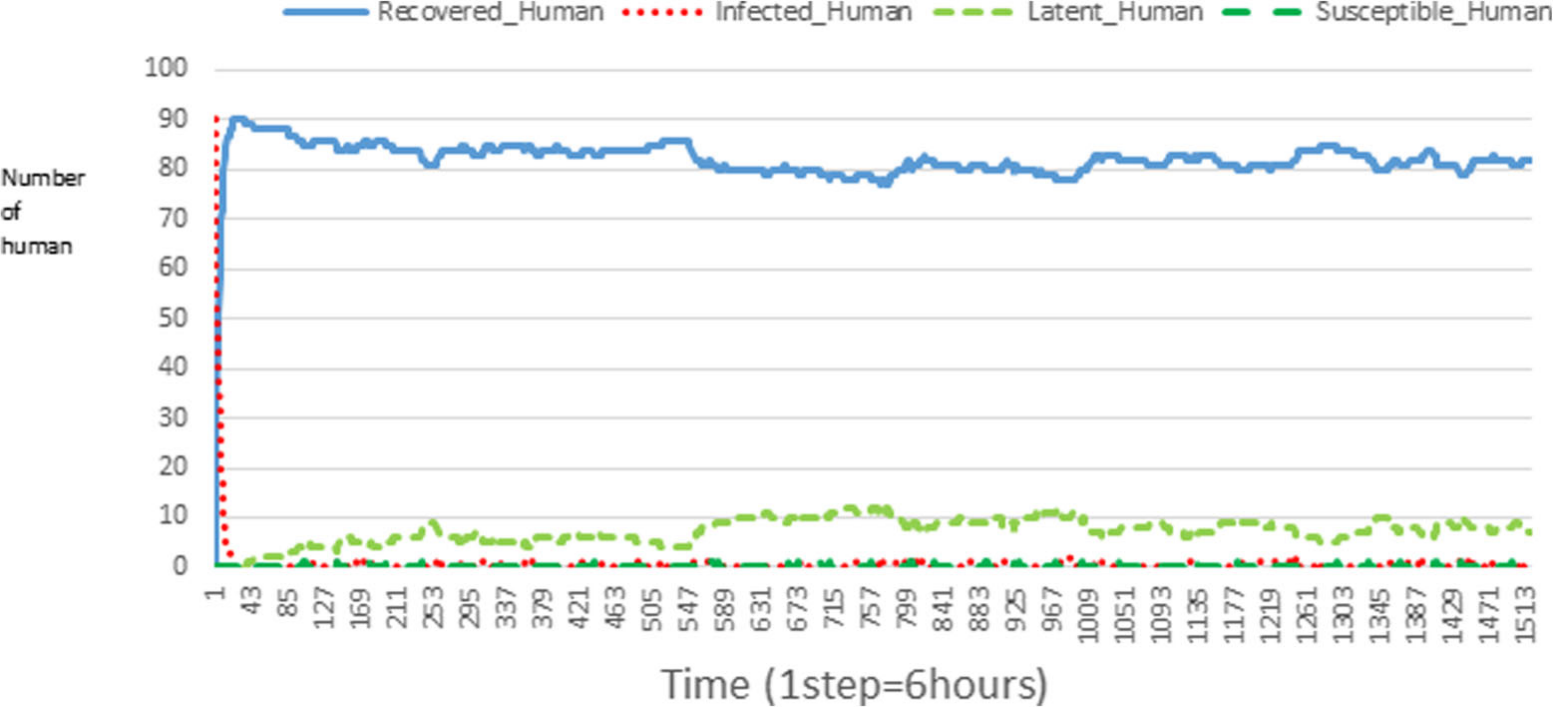
Human population: Evolution of susceptible, latent, infected, and recovery. The initial conditions are: number of infected human = 90, Number of AH = 15 *S*_*h*_ = 0, *E*_*h*_ = 0, *I*_*h*_ = 90, *R*_*h*_ = 0, *S*_*va*_ = 10, *S*_*vr*_ = 10, *E*_*va*1_ = 5, *E*_*va*2_ = 5, *E*_*va*3_ = 5, *E*_*vr*1_ = 5, *E*_*vr*2_ = 5, *E*_*vr*3_ = 5, *E*_*vr*4_ = 5, *I*_*va*_ = 10 and *I*_*vr*_ = 10

**Fig. 23:**
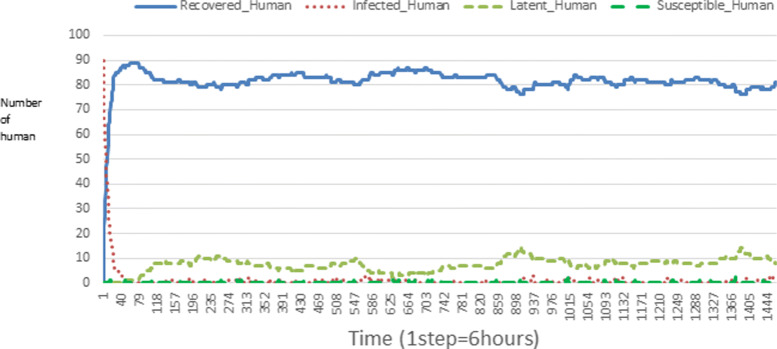
Human population: Evolution of susceptible, latent, infected, and recovery. The initial conditions are: number of infected human = 90, Number of AH = 30 *S*_*h*_ = 0, *E*_*h*_ = 0, *I*_*h*_ = 90, *R*_*h*_ = 0, *S*_*va*_ = 20, *S*_*vr*_ = 20, *E*_*va*1_ = 10, *E*_*va*2_ = 10, *E*_*va*3_ = 10, *E*_*vr*1_ = 10, *E*_*vr*2_ = 10, *E*_*vr*3_ = 10, *E*_*vr*4_ = 10, *I*_*va*_ = 20 and *I*_*vr*_ = 20

Figures 9, 10, 11, 12, 13, 14, 15, 16, 17, 18, 19, 20, 21, 22, 23 show the cases where there are HAs and mosquitoes in the environment. We can observe in these figures that the infection curve is never at 0. This is what explains the fact that the incidence is always different from 0 in these cases, hence the existence of malaria in the environment. Moreover, unlike the cases where there is no HA, we can observe that the human recovery curve is always increasing. This simply means that a lot of people have become infected at least once. Besides, since the death rate is low, most infected humans become recovered.

**Fig. 24:**
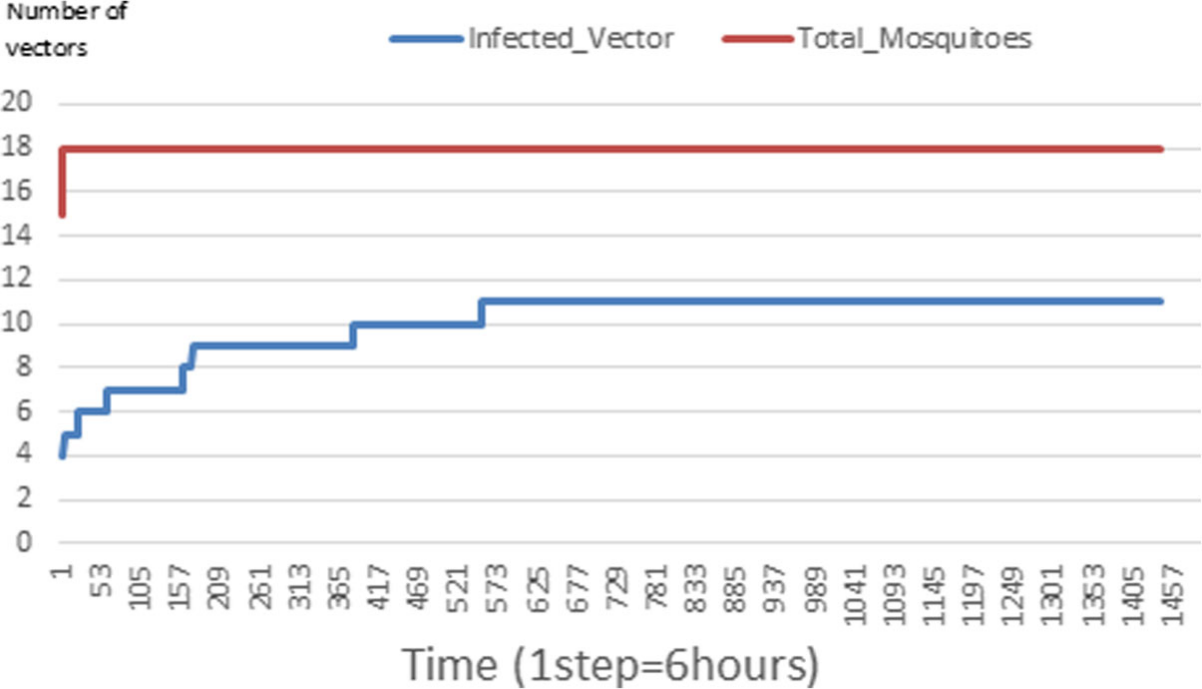
Vector population: Evolution of infected and total Vector. The initial conditions are: *S*_*h*_ = 40, *E*_*h*_ = 30, *I*_*h*_ = 0, *R*_*h*_ = 20, *S*_*va*_ = 2, *S*_*vr*_ = 2, *E*_*va*1_ = 1, *E*_*va*2_ = 1, *E*_*va*3_ = 1, *E*_*vr*1_ = 1, *E*_*vr*2_ = 1, *E*_*vr*3_ = 1, *E*_*vr*4_ = 1, *I*_*va*_ = 2 and *I*_*vr*_ = 2

**Fig. 25:**
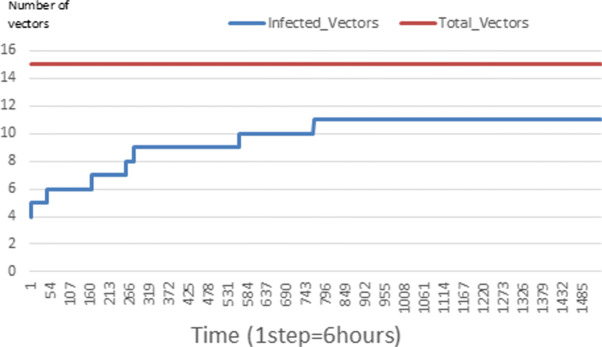
Vector population: Evolution of infected and total Vector. The initial conditions are: *S*_*h*_ = 39, *E*_*h*_ = 30, *I*_*h*_ = 1, *R*_*h*_ = 20, *S*_*va*_ = 2, *S*_*vr*_ = 2, *E*_*va*1_ = 1, *E*_*va*2_ = 1, *E*_*va*3_ = 1, *E*_*vr*1_ = 1, *E*_*vr*2_ = 1, *E*_*vr*3_ = 1, *E*_*vr*4_ = 1, *I*_*va*_ = 2 and *I*_*vr*_ = 2

**Fig. 26:**
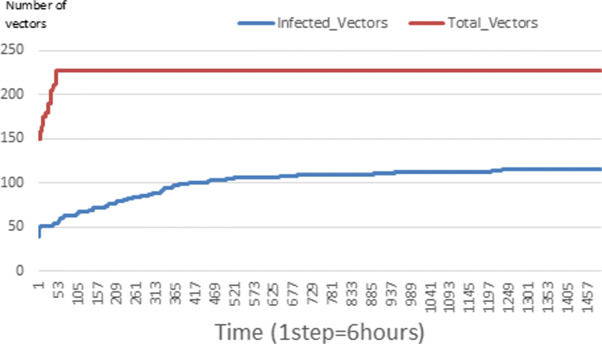
Vector population: Evolution of infected and total Vector. The initial conditions are: *S*_*h*_ = 39, *E*_*h*_ = 30, *I*_*h*_ = 1, *R*_*h*_ = 20, *S*_*va*_ = 20, *S*_*vr*_ = 20, *E*_*va*1_ = 10, *E*_*va*2_ = 10, *E*_*va*3_ = 10, *E*_*vr*1_ = 10, *E*_*vr*2_ = 10, *E*_*vr*3_ = 10, *E*_*vr*4_ = 10, *I*_*va*_ = 20 and *I*_*vr*_ = 20

**Fig. 27:**
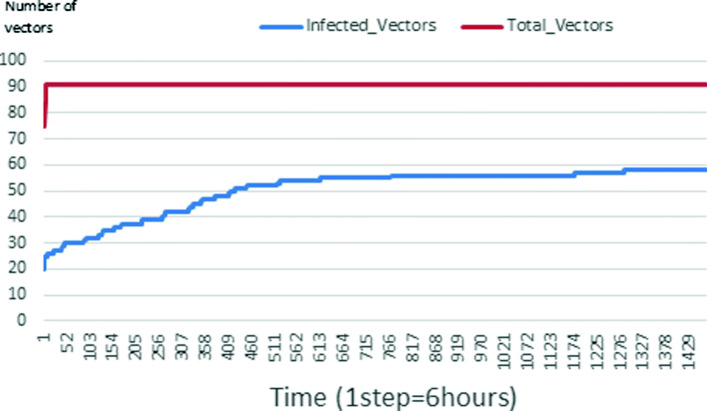
Vector population: Evolution of infected and total Vector. The initial conditions are: *S*_*h*_ = 31, *E*_*h*_ = 25, *I*_*h*_ = 9, *R*_*h*_ = 25, *S*_*va*_ = 20, *S*_*vr*_ = 20, *E*_*va*1_ = 10, *E*_*va*2_ = 10, *E*_*va*3_ = 10, *E*_*vr*1_ = 10, *E*_*vr*2_ = 10, *E*_*vr*3_ = 10, *E*_*vr*4_ = 10, *I*_*va*_ = 20 and *I*_*vr*_ = 20

**Fig. 28:**
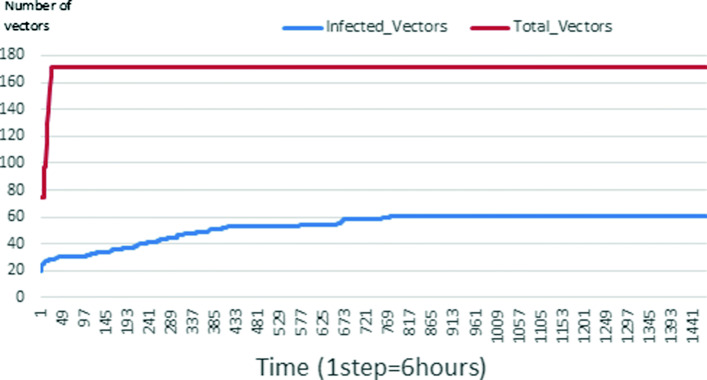
Vector population: Evolution of infected and total Vector. The initial conditions are: *S*_*h*_ = 15, *E*_*h*_ = 15, *I*_*h*_ = 45, *R*_*h*_ = 15, *S*_*va*_ = 10, *S*_*vr*_ = 10, *E*_*va*1_ = 5, *E*_*va*2_ = 5, *E*_*va*3_ = 5, *E*_*vr*1_ = 5, *E*_*vr*2_ = 5, *E*_*vr*3_ = 5, *E*_*vr*4_ = 5, *I*_*va*_ = 10 and *I*_*vr*_ = 10

**Fig. 29:**
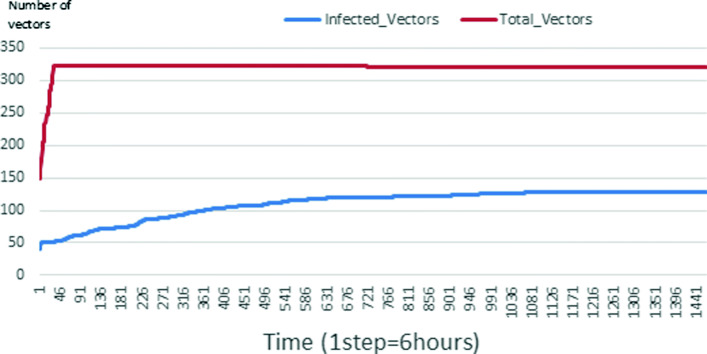
Vector population: Evolution of infected and total Vector. The initial conditions are: *S*_*h*_ = 15, *E*_*h*_ = 15, *I*_*h*_ = 45, *R*_*h*_ = 15, *S*_*va*_ = 20, *S*_*vr*_ = 20, *E*_*va*1_ = 10, *E*_*va*2_ = 10, *E*_*va*3_ = 10, *E*_*vr*1_ = 10, *E*_*vr*2_ = 10, *E*_*vr*3_ = 10, *E*_*vr*4_ = 10, *I*_*va*_ = 20 and *I*_*vr*_ = 20

Figures 24, 25, 26, 27, 28, and 29 show the population of vectors. These figures show that the number of infected humans depends on the growth of the number of mosquitoes in the environment. Indeed, if there are fewer infected humans in the environment, this means that mosquitoes are not able to take their blood meal properly. If mosquitoes fail to feed on the blood meal, it also means that their reproduction will also be reduced.

### Our results and those of the field

The simulation which corresponds to the normal situation in the field is when we destroy 0% of AH meaning the number of AH is equal to 30. So, when we simulate without destroying the AH, we find almost the same prevalence and incidence as those in the field. Thus, field data show that the prevalence is equal to 1.999% and the incidence is equal to 1.938%. When the number of AH is equal to 30, the prevalence varies from 1.159% (corresponds to the case that the initial number of infected is 0) to 2.057% (corresponds to the case that the initial number of infected is 90). Incidence varies from 0.936% to 1.145% (corresponds to the case that the initial number of infected is 0 90 respectively).

Noted that The prevalence and incidence in the grouping are low because the data was collected on the basis of patients attending the health center in the area. It should be noted that in African grouping, population doesn’t visit health centers regularly because they prefer traditional treatment.

### Impact on the wealth of infected mosquito

Mosquitoes are more numerous when there is more AH in the environment. This is explained by the fact that the resources available for the development of mosquitoes are necessary. On the other hand, when there are few AH, mosquitoes no longer have enough resources for their development. For example, we can see in Fig. 24 that throughout the simulation, the total number of mosquitoes does not exceed 18 and the number of infected mosquitoes does not exceed 11. This is explained by the fact that in the environment, there are no infected humans and the initial number of mosquito is only 5. So the probability of infected mosquito is low because all humans are healed. The same thing is seen in Figs 25 and 27. Worse, when there is no AH in the environment, we do not find any infected individuals. On the other hand, Figures 26, 28, and 29 show that mosquitoes are abundant in the environment. Figure 29 for example shows that the total number of mosquitoes can exceed 300. This can be explained as follows: as there are more than 15 AH and consequently more than 75 mosquitoes at the beginning, then there is a high chance that certain mosquitoes lay and develop their eggs. Indeed, even if in the model mosquitoes have many obstacles (death, death due to activity), some will still obtain resources for egg-laying and development.

Table 2 shows that the entomological inoculation rates (EIR; the number of infected mosquitoes divided by the number of persons) increases in the same way as incidence and prevalence.

**Table 2:** The evolution of prevalence, prevalence and entomological inoculation rates (EIR; the number of infected mosquitoes divided by the number of persons) when initial population is 20

Aquatic habitat reduction (%)	Remaining habitats (N = 30)	Malaria incidence	Malaria prevalence	EIR	Initial number of infected person
00	30	0,965	1,47	115,97	20
10	27	0,928	1,418	100,71	20
20	24	0,880	1,249	90,81	20
30	21	0,680	1,108	78,31	20
40	18	0,575	0,895	64,31	20
50	15	0,477	0,703	57,09	20
60	12	0,303	0,443	43,50	20
70	9	0,184	0,279	33,14	20
80	6	0,118	0,186	22,29	20
90	3	0,022	0,037	10,34	20
100	0	0,015	0,028	0	20
100	0	0	0	0	00

### Impact on the transmission of malaria

The transmission of malaria is strongly influenced by the presence of AH in the environment. Since the presence of AH attracts mosquitoes, more there are AH more mosquitoes exits. Thus, the incidence of malaria also changes with the number of individuals infected. Let us observe for example Figs. 19 and 20 then 28 and 29 (figures which describe respectively the same initial scenarios). We see that there are a lot of infected individuals and high malaria incidence.

### Effect of the destruction of aquatic habitats

The Table 2 presents malaria incidence and prevalence when the initial number of infected humans is 20 and 0. The table shows that more AH are destroyed, more incidence and prevalence tend more and more towards 0. On the other hand, when there is no AH and infected humans exist in the environment, the incidence and prevalence are evaluated only on those initially infected humans because this number of infected remains constant until the end of the simulation. Finally, if there are no AH sites and no infected humans in the environment, the incidence and prevalence are 0.

### Evolution of individual class according to the destruction rate of aquatic habitat

Looking at Figs 4, 5, 6, 7, and 8 in Presentation of some curves section, when all AH are destroyed, the population evolution is normal. Indeed, these figures show that after a certain time, the entire human population tends to be susceptible. On the other hand, even if there is only one AH in the environment, most humans living in that environment are recovered. This means that every human has at least once been achieved with malaria. This can be seen through Figs: 9, 10, 11, 12, 13, 14, 15, 16, 17, 18, 19, 20, 21, 22 and 23 of Presentation of some curves section.

### Our results and earlier studies

The peculiarity of our study is to show that the partial environment treatment (like TSR) of HAs does not help in the control of malaria. Recent studies tend to show the opposite. Indeed, in their studies, Gu *et al* [4] found very good results in proving that TSR is better than the global treatment of the environment. However, they could have found other things if certain parameters and/or assumptions had been taken into account in their studies [5]. Other authors notably criticized them for not properly applying the disposition of landscapes using absorbing and non-absorbing boundaries, the disposition of houses, and AH and also for not doing several simulations [5]. Thus, Arifin et al. Have solved this problem in [5]. In their studies, Arifin et al. ([5]) used a landscape generator tool and replicated 1,800 simulations using non-absorbing and absorbing boundaries. But, except that the aim of the work of Arifin et al. was not to show that the overall treatment is better than TSR. They just enunciate this situation in their work without providing concrete proof for it. Because of this, we show this with real proof.

## Discussion

Three important results emerge from our work. First, treating just a few aquatic habitats in one environment and leaving others behind is not helpful. Indeed, whether we use the targeted reduction technique or the overall reduction technique with 2 km of perimeters (as in recent studies [4]), we just reduce the incidence of malaria but we do not return it to 0. This means that malaria will go away for a certain time but come back later if the treatment is not done regularly. Our simulations show that it is when we destroy all the AH in an environment that we can greatly reduce the development cycle of the mosquito, hence the eradication of malaria. Indeed, by destroying AH, the mosquito no longer has a place to lay the eggs, lava development, and a place to rest. However, large-scale treatment poses a problem of resources, cost, and timing. If you want to do a large-scale treatment, you need a lot of materials to do it which will give rise to a lot of expenses. The timing is important here because if the overall treatment is not done simultaneously, there is a good chance that mosquitoes from an untreated area will migrate to areas already treated. But these problems could still be addressed using new environmental management mechanisms such as the use of drones. This also poses another problem: the effect of the products to be used on the hosts (humans and animals).

Second, it is important to destroy only mosquitoes that cause malaria. Destroying all mosquitoes can have a lot of impact on the ecosystem as many species feed on mosquitoes [7]. Starting from the fact that not all mosquitoes species are blood-sucking (just 6% [12]), we could thus eliminate only those who seek the host for their blood meal. However, the question of adopting a product that kills only certain species of mosquitoes without a primary and/or secondary effect on the other species remains.

Third, sensitize the population and find ways to sanitize these areas can be the main control measure. Using the two results of the struggles listed above will be useless if the populations continue to follow their habits. Indeed, if the population continues to pour wastewater close to homes, this could allow mosquitoes to continue their development (human and aquatic habitats are available). In addition to sensitization, it will also be necessary to think about cleaning up their environment by channeling wastewater. However, there is a major challenge in the techniques of sensitization and sanitation. Indeed, most African areas, especially those in the South Sahara, are enclosed [3] and difficult to access [3], hence the issue of sensitization and sanitation remains unresolved. But, a draft of a solution can be brought within the sensitization in particular by using the means of communication which exist more and more nowadays (example: social networks, proximity radio, communicating to the population through their leaders traditional, …). This communication or sensitization can also be used to show the simple and easy techniques of wastewater sanitation and/or pipes to the populations.

In this model, the distance that mosquito covers between its aquatic habitat and a human house played a major role in the mosquito’s ability to obtain resources. Unfortunately, the distance at which mosquitoes can find a certain resource is poorly understood. Recent studies show that mosquito can fly 200-400 m per day. Also, a mosquito can fly up to 10-12 km during its lifetime. From these results, our model shows that if we remove just a few AH, an infected mosquito can contaminate an environment having a surface which range is more than 10 *km*^2^ of its AH. Thus, the disease can be prolonged in the environment if only a few AH are destroyed. This result suggests that forbearing mosquitoes from their resources can severely limit their development and growth around AH.

We have found that resource searching is related to a mosquito flying over distance. Indeed, after mating, the mosquito seeks humans for the blood meal to mature these eggs. After the blood meal, mosquito looks for a place to lay and develop its eggs. Knowing that mosquito can daily travel 200-400 m, destroying the AH would make that mosquito couldn’t easily find a resource. So, increasing the flying over’s distance of mosquito out of their resources for egg-laying and maturation of eggs could greatly slow down or completely reduce the number of mosquitoes. Mosquito flight and its foraging are influenced by the disposition of resources in the environment.

Given the relentless fight against malaria in Africa, it is important to propose a new direction in the techniques of the fight. It should be noted that our fight does not fall only on the fight by larvicides, but also on the environmental sanitation through the pipeline and sensitization of the populations on the management of domestic waste and wastewater from their household. Although treating water may help to reduce the development of hematophagous mosquitoes, but does not help the ecology. To help humans on the fight against malaria and ecology in the preservation of certain species, it is important to operate only on hematophagous (that feed on blood) mosquitoes since they are only a minority (6%) [12]. So to be able to operate on these species only, we suggest to the governments to sensitize their populations on the management of their environment. This can be done for example by using the means of communication that exist today to give the building plans even to those living in the deep villages. Also, the canalization of wastewater used by households and domestic waste can be resolved by proposing the methods which use the means adapted to Africa. We can also focus studies on products against mosquitoes that would only operate on hematophagous species without having effects on humans, other species of mosquitoes, animals, and other insects.

Agent-based models are used to understand complex phenomena such as the transmission and spread of malaria. We use this to understand the dynamics of malaria transmission in a grouping. Since the models often give a lot of uncertainties, we took several parameters into account to bring nearer reality and make models to be more robust. Also, we use the data field in other to not go away from reality.

## Conclusion

In this paper, ABM was proposed to assess and examine the impact of mosquito’s AH destruction program and also bring out guidelines for LSM by AH destruction. We have noted that several studies have worked in this domain and find good results but neglecting certain aspects or parameters in their model. We have therefore solved this problem by taking in our model many parameters. We took into account the questing-resting to represent the number of infectious bites in our ABM model which had not yet been carried out until now. To achieve this, we used the parameters of some mathematical and ABM models. We also took into account: the different stages of the disease, the life cycle of the mosquito, the daily activities, heterogeneous landscapes, status, and movement. It emerges from this study that makes global destruction is great than TSR. Also, the use of many parameters in the same model makes the model more robust. The main limitation of our study is that it is not robust enough because some control parameters (such as the use of the INT for example) were not considered. Also, the fact of not using the means of protection limits the observation of the movements of mosquitoes as it happens in reality. To make work even more robust and efficient, in future work, we provide to include the use of insecticide net treated associate with the aquatic habitat destruction in other to well understand the behavior of individuals (mosquitoes and host). other parameters will be added also to the model like the activity of humans for example.

## Data Availability

The datasets used and/or analysed during the current study are available from the corresponding author on reasonable request. All data generated or analysed during this study are included in this published article (See the list of figures).

## Abbreviations

ABM: Agent-based model
AH: Aquatic Habitats
AHM: Aquatic Habitat Management
EM: environmental management
ITN: insecticide-treated nets
LSM: larval source management
SEI: susceptible-latent-infected
SEIRS: susceptible-latent-infected-recovered-susceptible
TSR: targeted source reduction
Qgis: Quantum GIS (geographic information system)
WHO: World Health Organization
